# Progress in Medicine

**Published:** 1898-08-20

**Authors:** 


### Progress in Medicine.

### FEVERS.

(*Continued from page 338.*)

Typhoid (*continued*). — Petruschky[1] found typhoid bacilli in the urine in only three cases out of 50, but they were present in enormous numbers, appeared suddenly, and remained after convalescence had taken place. Hence the importance of disinfection of the urine up to a late stage in the disease. One of Petruschky's nurses actually took the disease through infected urine, which had accidentally contaminated a drinking vessel. It is not clear what is the cause of this sudden appearance of large numbers of bacilli in the water—whether it occurs when the blood contains an unusual number, or after immunity has been attained, or merely after some lesion of the kidney has permitted their excretion. If the bacilli are often not to be found in the urine till late in the disease, it is plain that we cannot hope to have a means of diagnosis in this phenomenon. Another symptom, discussed by Quentin,[2] is a yellow pigmentation of the palms and soles, which has not hitherto received much notice. He refers this to some change in the nutrition of the skin through elimination of toxic products, and has noticed a similar slight change in rheumatism and tuberculosis, but not to the same degree as in typhoid. Osler[3] insists that the disease is systemic, and not localised in the intestine, for not only are cases found without ulceration, but he has seen one without any affection even of the intestinal lymphatics. Hence he is opposed to the use of intestinal antiseptics and salines as a supposed means of removing the disease from its seat. Nichols and Heenan[4] in discussing a case of typhoid without intestinal lesions, find there are nine such cases reported where Eberth's bacillus was present. They divide them into (*a*) typical typhoid minus the ulcerations, (*b*) spleno-typhoid, and (*c*) the nervous type with intoxication, to which class their own case belonged. As an explanation of the phenomena, they suggest either that the infection entered by some unwonted method, *e g.*, by the lungs, or that certain ptomaines in the intestinal tract bring about a local immunity, or finally, that toxins from other germs bring about a similar local protection. Chiari and Krauss also consider such cases as typhoid septicæmia, and have demonstrated the presence of bacilli in three instances. Mallory[5] believes that lesions when present are produced by the absorption of a mild poison, which is absorbed partly from the blood and partly from the intestinal tract. The absorption takes place both through the lymphatics and through the capillaries. The lesions in the mesenteric glands are caused by absorption through the lymphatics, while those in the rest of the body depend primarily on the toxins in the general circulation.

A little noticed complication of the disease is erysipelas, which Gerente finds[6] in about one case in every sixty-four, most often in females, and generally after the third week. The facial form is specially fatal, but in other types the typhoid symptoms usually improve when an attack of erysipelas comes on. The frequency of typhoid in the aged is shown by M. Manges[7] to be greater than is usually thought. He quotes Osler as recording 1·3 per cent. of patients over 60 years, and one of the Johns Hopkins' cases without intestinal lesions was 68. He himself has recently treated five over 60 years, and notices the frequently insidious onset of the complaint in aged persons, together with an irregular type of fever, and marked bronchitis and asthenia. In New York the proportion of deaths from typhoid in persons of 65 years and over was 2 6 per cent. of the whole. The diagnosis, apart from the Widal reaction, is difficult, and the prognosis unsatisfactory, but it is important to employ tonic treatment, and to watch the heart with more than ordinary care. Sidney Martin,[8] in the Croonian lectures, divides bacterial poisons into (*a*) the secretions of the organisms, (*b*) the products of their digestive action, (*c*) their final non-proteid product or alkaloid, and (*d*) the poisons in the bodies of the bacilli; but he finds no evidence that this last poison differs in the case of typhoid from the other extra-cellular ones. A marked effect of the injection of typho-toxin into animals is the production of diarrhœa, as well as a loss of body weight and degeneration of the heart muscle. The action of the poisons of the bacillus coli and the bacillus enteriditis of Gartner is very similar to that of the typhoid one. It has been also often laid down that typical typhoid fever cannot be communicated to animals; but Fraenkel and Simmonds[9] have lately shown it possible, without injections, by simply feeding rabbits on a copious and continued diet of lettuce soaked in water containing the bacillus. Three of their animals suffered from typical attacks of typhoid, with fever, diarrhœa, Widal's reaction, ulceration of Peyer's patches, and enlarged spleen, while cultures of the bacillus were made from their bodies. Similar results were obtained with white rats. These results are not only important for experimental work, but form an epoch in the history of the discovery of the bacillus. For now, at last, is the proof complete, according to Koch's canons, that the bacillus is the specific cause of the disease. The value of antityphoid vaccination is shown by J. S. Tew[10] from the results at the Kent County Lunatic Asylum, where an outbreak occurred. Not one of those vaccinated, 84 in number, took the disease, while 16 of the 120 unvaccinated who lived under similar conditions were seized by it. It should be added, however, that after a certain date other precautions were taken, so that the entire success may not be due to vaccination. The injections caused considerable malaise, but most of those who underwent it were well in three days, except for a stiff and painful arm. Tchistovitch[11] notices that the Widal reaction in typhoid is feeble in severe cases until the late stages of the disease, while it is well marked when the disease runs a favourable course. From this and various experiments he regards this power of the serum as a useful quality tending to produce immunity. Courmont[12] regards it as a protective reaction of the organism during the period of infection, but notices that in the early stages of the disease the serum of typhoid patients actually favours infection. Later on its state changes, and we find an immunising power present. Still from the beginning there is an attenuating power in the serum, which is proportional to the amount of clumping produced in Widal's test. Thus he would recognise three effects of the serum of the patient on the bacillus—first, one favourable to its multiplication, then one protective against it, and throughout an influence which lessens its virulence. The continuance of the first state may be the cause of relapses. It has been stated by Freyer that healthy natives of India give a positive reaction to Widal's test, that they are practically immune to typhoid, and that both these phenomena are due to the universal incidence of the disease during their infancy. W. C. Brown[13] denies all these statements. He found no positive reaction in 15 persons of various castes whom he tested. Moreover, the reaction has been shown by many observers to disappear soon after an attack. Hence, if it occurred in adult Indians it could not be due to attacks in their infancy. The error may have arisen from the rapidity with which "false clumping" occurs owing to the climate. Only a short period should be allowed for reliable results. Finally, he remarks that the immunity from typhoid is not so great as is thought. In the absence of post-mortems many cases are set down to malaria and other causes. Indeed, he believes that the Eastern tropics are the birthplace and source of typhoid fever. Sheridan Delépine[14] has employed the Widal test in 841 cases, and remarks that the post-mortem results have, when obtained, always been in agreement except in one instance of doubtful character, nor has he found any disease in which a true reaction, like that of typhoid, is present. It is absent, indeed, in jaundice, and in the blood of the placenta and of the fœtus, where some observers believe it occurs. Gilman Thompson[15] looks with only modified approval on the test, contrary to the general opinion. He claims that it is uncertain in about 23 per cent. of the cases, which are just those where there is the greatest doubt from clinical evidence, and does not apparently regard it as much more reliable than the diazo reaction. Jaundice in typhoid is exhaustively discussed by Da Costa,[16] who finds it is due to the blood affection and alterations produced by the toxins on the cells of the liver, sometimes to gross changes induced there, and only occasionally to catarrh of the ducts. The more frequent of these causes is the blood infection, but it may result also from inflammation of the gall-bladder. The symptom is a grave one, and in 52 tabulated cases he finds 33 deaths.

[1] Brit. Med. Jour., June 25. [2] Brit. Med. Jour., June 11. [3] Med. Rec., Feb. 26. [4] Amer. Jour. Med. Sci., May. [5] New York Med. Jour., May 28. [6] Clin. Jour., Jan. 26. [7] Med. Rec., Feb. 26. [8] Brit. Med. Jour., June 18. [9] Brit, Med. Jour., Mar. 18. [10] Lancet, Mar. 19. [11] Brit. Med. Jour., Mar. 12. [12] Brit. Med. Jour,, May 21. [13] Brit. Med. Jour., Mar. 12. [14] Lancet, Feb. 19. [15] Med. Rec., June 28. [16] Amer. Jour. Med. Sci., July.

### DISEASES OF THE CIRCULATORY SYSTEM.

**Acute Inflammatory Disease of the Heart and Endocarditis.**—Powell[1] lays great stress on the supreme importance of absolute rest in bed in the prevention as well as in the treatment of rheumatic endocarditis. Salicylate of sodium materially lessens the liability to endocarditis in acute rheumatism. If it sets in, alkalis and salicylate of sodium should be given. Local treatment, whether by blisters, leeches, cold, or warmth, seems to have little influence. Digitalis is useless in acute inflammatory heart affections, and aconite, with certain exceptions, harmful. In peri- and myo-carditis, especially when there is pain and irregular rhythm and a tendency to tumultuous action and heart failure, small doses of opium at frequent intervals are of great service; they calm irritation and restlessness and secure more heart rest. When the acute stage passes away arsenic, strychnine, and iron are useful; five-grain doses of mixed iodide of soda and ammonia three times a day seem to be of some advantage in hastening resolution of the inflammatory thickenings. If there be quick or irregular cardiac rhythm, digitalis may be given, but not if this be due to inflammatory irritation, which is indicated by a raised temperature and a soft, low-pitched murmur. Perfect sanitation during treatment is very desirable in connection with the possibility of ulcerative endocarditis, and in convalescence the seaside is preferable to the country. In using digitalis it should be remembered that each form of valvular disease has its own type of pulse, and digitalis should be given to maintain this type. In aortic regurgitation, for instance, it should be given when an irregularly feeble pulse and right heart embarrassment give evidence of ventricular failure. When, however, there is reason to think that the ventricle is only overworked, owing to contraction of peripheral vessels, as in gouty conditions, mercurials and salines are more useful than digitalis. It is never necessary to lessen the work of the hypertrophied heart by aconite. In mitral stenosis digitalis in early stages may cause constriction of the arteries, and do harm. When there is cardiac failure, the first indication is to unload the venous system by intestinal or hepatic derivatives and a restricted diet. But if the pulse remain quick after such treatment digitalis is called for. In mitral regurgitation digitalis should not be used unless there is irregularity of the pulse, with evidences of dilated ventricle and failing urine excretion. It should then be given until the pulse becomes slow. The sickness which occasionally supervenes on digitalis may be sometimes prevented by occasional mercurials. A tumbler of hot water, taken at intervals, may at times be given with advantage.

**Heart Complications in Diphtheria.**—Hibbard[2] finds that less than one-half of the cases in which the pulse rate exceeds 100 recover. The pulse rate and mortality appear to be very much in a direct ratio to one another, and recovery is comparatively rare with rates above 150. Extreme slowness of the pulse is less significant, but in children bradycardia does at times presage evil. Variations of rhythm and volume occur in about 10 per cent. of cases, and are a useful premonition of cardiac complications. A systolic murmur at the apex of the heart is heard in about one case in 10. This is commonly due to mitral insufficiency, caused by dilatation, but in rare instances to an endocarditis of diphtherial origin. The murmur is not itself a serious matter, but if an increasing area of cardiac dulness and accentuation of the pulmonary second sound indicate a dilated ventricle the outlook is less favourable.

**Relaxation of the Heart.**—Feilchenfeld[3] points out that after fatigue the heart is in a temporarily relaxed condition, similar to that of the skeletal muscles after severe exertion. For instance, after wrestling the heart may be temporarily dilated and contract with diminished force, as shown by the pulse. This temporary and physiological relaxed condition of the organ merges by intermediate degrees into one of actual dilatation. Three phases of relaxation may be distinguished: (1) A premonitory stage characterised by palpitation, excitability of the heart's action, feeling of fatigue, and slight anxiety. Cases of this kind should not be regarded as simply nervous. As etiological factors may be mentioned rapid growth at puberty, sexual excesses, physical and mental overwork, anæmia, alcohol and nicotine, previous illness, and premature old age. (2) The first stage of actual relaxation. (3) Actual dilatation, which has been long recognised. But the earlier stages above mentioned exist and should be also recognised, so that the condition may be opposed in time. Palpation is more important than percussion in estimating the size of a relaxed heart; this is best done when the patient is in the leaning forward attitude.

**Exercises in the Treatment of Heart Disease.**—Powell[4] says that massage stimulates the circulation in the muscles and promotes the passage of lymph through the lymphatic vessels; the metabolism of the body is thus maintained in those who from any cause are unable to take active exercise. The treatment is not to be advised in acute heart affections, whilst it may be of service in the convalescent stage of acute heart affections and in combating the tendency to stagnant circulation in those who are disabled by chronic heart disease, or who, bed or sofa ridden from any other cause, tend to impairment of heart nutrition and suffer from chilly extremities, feeble pulse, and torpid digestion.

Resistance exercises, often called Schott or Nauheim exercises, are governed by more complex considerations. Their effect may be said to be a stimulation of the heart's action, with some steadying effect and increased completion of systole, an improved circulation through the coronary vessels, and an increased mobility of the blood by its readier passage in greater bulk through the muscles, thus relieving stagnation in the great internal organs, especially on their venous side.

Resistance exercises are especially adapted for the initial treatment of cases of flabby, irritable heart, found in people of venous plethora; again, they are valuable in cases of chlorosis with dilated heart, also in commencing failure of the heart in chronic valve lesions. All cases of acute endocarditis, whilst there is any trace of activity of lesion remaining, are still more unsuitable for this than for massage treatment. Cases of advanced cardio-vascular changes of the nature of sclerosis, and particularly when associated with granular kidneys, are absolutely unsuited. In cases of introspective people with neurotic hearts, the treatment is best avoided; these patients require a better occupation than that of indoor gymnastics. The exercises are unfitted for cases of tachycardia and exophthalmic goitre. The strong brine and aërated baths of Nauheim and other places are of much service in some circulatory disorders, and perhaps are most suitable for cases of chronic rheumatism and gout, associated with high arterial tension and secondary cardiac disturbances. They may also be used in cases of functional excitement of the heart's action in connection with quiescent or only imperfectly-compensated valvular affections.

**Use of Röntgen Rays in Diagnosis.**—Schott,[5] of Nauheim, has published some skiagrams showing the effect of baths and exercises on the size of the heart. He remarks that, measuring the heart muscle postmortem, which has served till now as a basis for judging of the increased size of the heart, is wholly unreliable. Hence many men, though convinced of the therapeutical effect of the treatment, have been unable to account satisfactorily for the immediate effect of the baths and exercises, and some even have attributed the undeniable apparent reduction of its size to an increased over-lapping by the lungs. This view, however, is easily refuted by observation of the fact that the apex beat works visibly and palpably upward and inward, whilst the diaphragm rises simultaneously, and the margins of the lungs remain unchanged. Observations by means of the Röntgen rays have been conducted both by photographic plates and by the fluoroscope. Both methods demonstrate that systematic exercises and baths diminish appreciably the size of the heart. The immediate effect of the bath is not quite so marked as after the exercises, but it has the advantage of lasting longer; whilst, in favour of the exercises, it may be stated that they can be used repeatedly in one day and without regard to place.

Examination by means of the Röntgen rays also easily demonstrates how over-straining produces dilatation, *i.e.*, weakening of the organ. Experiments were made by causing people to wrestle until the breath became very short, and taking photographs before and after. Schott was also able to demonstrate that bicycling on bad roads or against a strong wind easily produces dilatation of the heart. He has thus been able to prove statements for whose truth he has been contending for many years.

[1] Powell, Brit. Med. Jour., April 2, 1898. [2] Hibbard, Boston Med. and Surg. Jour., Jan 27 and Feb. 3, 1898. [3] Feilchenfeld, Berl. Klin. Woch., Feb. 28. 1898. [4] Powell, Brit. Med. Jour., April 9, 1898. [5] Schott, Med. Record, Mar. 26, 1898.

